# Differential Cognitive Functioning and Benefit From Surgery in Patients Undergoing Coronary Artery Bypass Grafting and Carotid Endarterectomy

**DOI:** 10.3389/fneur.2022.824486

**Published:** 2022-03-08

**Authors:** Kristiina Relander, Marja Hietanen, Juhani Rämö, Antti Vento, Irene Tikkala, Risto O. Roine, Perttu J. Lindsberg, Lauri Soinne

**Affiliations:** ^1^Division of Neuropsychology, Neurocenter, Helsinki University Hospital and University of Helsinki, Helsinki, Finland; ^2^Division of Cardiac Surgery, Heart and Lung Center, Helsinki University Hospital and University of Helsinki, Helsinki, Finland; ^3^University of Turku and Turku University Hospital, Turku, Finland; ^4^Department of Neurology, Neurocenter, Helsinki University Hospital and Clinical Neurosciences, University of Helsinki, Helsinki, Finland

**Keywords:** coronary artery bypass grafting, atherosclerosis, carotid endarterectomy, cognition, neuropsychological tests, executive functioning, coronary artery disease, carotid artery disease

## Abstract

**Background:**

Stenosing atherosclerosis in both coronary and carotid arteries can adversely affect cognition. Also their surgical treatments, coronary artery bypass grafting (CABG) and carotid endarterectomy (CEA), are associated with cognitive changes, but the mechanisms of cognitive decline or improvement may not be the same. This study was designed to compare the cognitive profile and outcome in patients undergoing surgical treatment for coronary or carotid disease.

**Methods:**

A total of 100 CABG patients and 44 CEA patients were recruited in two previously reported studies. They were subjected to a comprehensive neuropsychological examination prior to surgery and in the acute (3–8 days) and stable (3 months) phase after operation. A group of 17 matched healthy controls were assessed with similar intervals. We used linear mixed models to compare cognitive trajectories within six functional domains between the CABG, CEA and control groups. Postoperative cognitive dysfunction (POCD) and improvement (POCI) were determined with the reliable change index method in comparison with healthy controls.

**Results:**

Before surgery, the CEA patients performed worse than CABG patients or healthy controls in the domains of executive functioning and processing speed. The CABG patients exhibited postoperative cognitive dysfunction more often than the CEA patients in most cognitive domains in the acute phase but had regained their performance in the stable phase. The CEA patients showed more marked postoperative improvement in executive functioning than the CABG group in the acute phase, but the difference did not reach significance in the stable phase.

**Conclusion:**

Our findings suggest that anterior cerebral dysfunction in CEA patients impairs preoperative cognition more severely than global brain dysfunction in CABG patients. However, CEA may have more beneficial effects on cognition than CABG, specifically in executive functions mainly operated by the prefrontal lobes. In addition, the results underline that POCD is a heterogeneous condition and dependent on type of revascularization surgery.

## Introduction

Atherosclerotic disease is nowadays the leading cause of death worldwide (1). Its most severe consequences are stenoses in the coronary and carotid arteries. Whereas coronary arteries supply the heart that circulates blood in the whole body including the brain, the internal carotid arteries provide circulation specifically to the anterior parts of the brain and to the eyes. Atherosclerosis can lead to cognitive deterioration even before severe consequences such as acute myocardial infarction (AMI) or cerebral stroke. Chronic heart failure can result in global reduction of blood circulation in the brain (2) and 19% of coronary artery disease patients exhibit cognitive impairment before surgical treatment (3). In a similar vein, carotid artery stenosis is related to impaired cognition even before stroke or transient ischemic attack (TIA) (4, 5). These changes may follow either from hypoperfusion of the anterior parts of the brain leading to impaired cerebrovascular reactivity (6, 7) and gradual ischemic changes (8) or from showering of emboli from carotid plaque (9), ultimately leading to neuronal loss. To date, cognitive performance in atherosclerotic patients with a primary symptom-generating lesion in carotid or coronary artery have not been compared.

Both carotid and coronary artery stenosis can be treated surgically. In coronary artery bypass grafting (CABG), the blocked part of the artery is bypassed with a healthy artery or vein from the body. In carotid endarterectomy (CEA), the atherosclerotic plaque is removed surgically. Both surgeries have been associated with cognitive sequelae. According to a meta-analysis, postoperative cognitive dysfunction (POCD) was evident in about 43% of CABG patients shortly after surgery, 19% at 4–6 months, 25% at 6–12 months and 40% at 1–5 years after surgery, although prevalence was greatly influenced by methods used for classification of POCD (3). Regarding postoperative cognitive improvement (POCI), a few controlled studies have also showed evidence for improved cognition in some patients after CABG (10, 11). However, it seems that the cognitive benefits of CABG are transitory, as improvement shown 3 years after surgery (11) is lost in longer follow-up at 6 years (12). CEA, in contrast, has been associated with approximately similar rates of POCD and POCI: decline in 10–15% and improvement in about 10% of patients 1–3 months after the operation (13–19). At 6 months, decline in 22% of patients (20) and improvement in 33% of patients (21) has been reported.

Several risk factors for postoperative cognitive decline have been suggested. A recent meta-analysis identified preoperative depression, age, longer intraoperative intubation time and longer postoperative intensive care as risk factors for POCD after CABG (22). Also other surgery-related factors, such as using cardiopulmonary bypass, neurotoxicity of anesthetics, temperature management, embolic load, neuroinflammation, stress response, cerebral blood flow and glucose homeostasis, as well as patien*t-*related factors, such as neurovascular disease, lower education, lower preoperative cognitive function and female sex in older age have been suggested as risk factors for POCD after cardiac surgery (23–25). Specific mechanisms of POCD after CEA include complications such as intraoperative ischemia (26, 27), embolization (28) or postoperative hyperperfusion (29). Postoperative cognitive improvement after CABG has been suggested to follow from reduced need for medication, better physical health and quality of life (30). The pathways for improved cognition after CEA seem more specific: POCI after CEA has been shown to be associated with improvement of cerebral perfusion (31), metabolism (17, 18, 32) and cerebral vasomotor reactivity (33).

Postoperative cognitive impairment after CEA or CABG has been associated with lower quality of life (34), adverse long-term cognition (10, 35–37) and higher mortality (35, 38, 39). However, differences in postoperative cognitive outcome between CABG and CEA are yet to be investigated.

The aim of this study was to compare the cognitive profile and outcome in patients undergoing surgical treatment for carotid or coronary disease in two cohorts from previously reported studies. Based on our previous findings (10, 38) we hypothesized that carotid disease causing territorial cerebral hypoperfusion would be associated with a deeper and more localized cognitive preoperative deficit in CEA patients and correspondingly a more pronounced postoperative improvement, whereas CABG would be associated with a more general short-term postoperative decline.

## Materials and Methods

### Setting

This study was performed in the Helsinki University Hospital, Finland. The data comprised of patient groups from two studies described earlier: the CEA group (13, 38) and the CABG group (10). Both studies were approved by the ethical committee of the Helsinki University Hospital and performed according to the ethical standards of the Declaration of Helsinki. All participants gave their written informed consent to participate in the studies. The CEA data was collected between 20.2.1997 and 23.3.2000 in the Departments of Neurology and Cardiovascular and Thoracic Surgery, Helsinki University Hospital, Finland, and the CABG data between 2.4.1995 and 29.3.1996 in the Department of Thoracic and Cardiovascular Surgery, Helsinki University Hospital, Finland.

### Participants

Characteristics of the study groups are shown in Table 1. The CEA group was enrolled from a larger cohort, the Helsinki Carotid Endarterectomy Study (HeCES) (40) upon patient consent as well as availability of imaging facilities and personnel within the time frames defined in the protocol. The group consisted of 44 CEA patients who were independent, had no history of ipsilateral CEA or radiotherapy, no potential cardiogenic origin of emboli, no major psychiatric diseases requiring continuous treatment, and had a surgically accessible symptomatic (TIA or minor stroke, i.e., no need for inpatient rehabilitation or a deficit that would be expected to limit the neuropsychological assessment, *N* = 21) or asymptomatic (*N* = 23) unilateral carotid stenosis of at least 70 % in digital subtraction angiography (NASCET criteria). Of the patients, 20 were operated on the right side and 24 on the left side.

**Table 1:** Characteristics of the study population.

	**Controls (*N =* 17)**	**CEA patients (*N =* 44)**	**CABG patients (*N =* 100)**	***p-*value**
Age, years	63.6 ± 6.8	63.8 ± 8.8	60.4 ± 8.6	0.08
Sex, male	12 (71%)	28 (64%)	86 (86%)	0.008^**^
Education class				0.56
Basic level	7 (41%)	23 (52%)	44 (44%)	
Middle level	7 (41%)	16 (36%)	48 (48%)	
Higher level	3 (18%)	5 (11%)	8 (8%)	
Occupation class				0.57
Manual labor	4 (24%)	12 (27%)	39 (39%)	
Skilled manual	8 (47%)	20 (46%)	40 (40%)	
Non-manual	5 (29%)	12 (27%)	21 (21%)	
Diabetes	0 (0%)	8 (18%)	17 (17%)	0.86^*a*^
High blood pressure	0 (0%)	24 (55%)	55 (55%)	0.96^*a*^
Dyslipidemia	0 (0%)	26 (59%)	53 (53%)	0.50^*a*^
Smoking, pack years	N/A	27 (30.0)	13 (29.5)	0.009^a^^**^
Body mass index	N/A	27.1 ± 3.7	27.3 ± 3.6	0.81^*a*^
Depressive symptoms				
Baseline	2.0 (2.0)	4.0 (6.0)	4.0 (4.0)	1.00^*a*^
Acute phase	1.0 (2.8)	4.0 (7.0)	5.0 (5.0)	0.34^*a*^
Stable phase	1.5 (2.5)	4.0 (8.0)	3.0 (3.0)	0.27^*a*^
Congestive heart disease	0 (0%)	0 (0%)	6 (6%)	0.18
History of CEA	0 (0%)	0 (0%)	0 (0%)	1.00^*a*^
History of CABG	0 (0%)	8 (18%)	0 (0%)	0.000^a^^***^
History of AMI	0 (0%)	9 (20%)	53 (53%)	0.000^a^^***^
History of CABG or AMI	0 (0%)	12 (27%)	53 (53%)	0.004^a^^**^

*Data are presented as mean ± SD, median (interquartile range) or N (%). p-values are from Kruskal–Wallis test, Mann–Whitney U tests, independent samples t-tests, χ^2^ tests and Fisher's exact tests*.

a *The control group was omitted from statistical tests comparing risk factors, medical history and mood between groups*.

** *p < 0.01*,

*** *p < 0.001*.

*CEA, carotid endarterectomy; CABG, coronary artery bypass grafting; AMI, acute myocardial infarction; N/A, not applicable*.

The CABG group included 103 patients who went through an elective CABG. Three patients missed both postoperative assessments and were excluded from the study. The participants were independent in daily living and exhibited no neurological disability or cerebrovascular events within 6 months and no major psychiatric diseases requiring continuous treatment. A carotid ultrasound was performed prior to operation in order to preclude a significant carotid stenosis.

The control group included 17 healthy volunteers with no medications, no signs or history of cardiovascular or neurological morbidity, no excessive and long-lasting alcohol consumption, no major psychiatric diseases requiring continuous treatment, and no family history of neurodegenerative diseases. Age, sex, and educational and occupational level of the controls matched with both patient groups (Table 1). We rated education according to the Finnish educational system in three levels: basic level (compulsory education requiring 6–9 years of education), middle level (vocational training, matriculation examination and/or bachelor's degree requiring 8–15 years of education), or higher level (university level master's degree or higher requiring a minimum of 16 years of education). Occupational attainment was scored to three classes: physical or manual labor workers, skilled manual professionals, and non-manual white collar workers.

### Surgical Procedure

The CEA patients were operated on with standardized methodology under general anesthesia with routine hemodynamic and transcranial Doppler monitoring. Due to stump pressure, shunting was performed in three patients. A 1.5 Tesla magnetic resonance imaging of the brain was done on the day before CEA and approximately 4 days and 3–4 months postoperatively.

The CABG patients were operated on with a standard approach under general anesthesia with cardiopulmonary bypass. Ischemia time and cardiopulmonary bypass time were recorded. A membrane oxygenator was used, the perfusion pressure was kept between 50 and 80 mmHg, the nonpulsatile pump flow was kept at 2.4 L/min/m2, the and the core temperature was kept at 35°C.

### Neuropsychological Assessment

The patients were assessed at baseline (1–2 days before surgery), in the acute phase (CEA patients median 4 days after surgery, range 3–7; CABG patients median 7 days after surgery, range 6–8) and in the stable phase (CEA patients median 94 days after surgery, range 68–163; CABG patients median 93 days after surgery, range 80–112). The control group was assessed approximately at similar intervals (between first and second assessment median 5 days, range 4–7; between first and third assessment median 117 days, range 89–180).

Only tests used in both the CEA and CABG studies were chosen for this comparative study. We arranged the neuropsychological tests into six functional domains. Parallel memory test versions were used in repeated measurements in order to minimize learning effects. The neuropsychological test battery is show in Table 2.

**Table 2:** Neuropsychological test battery.

Learning	Auditory verbal learning test (10 words, sum of five trials) (41)
	Logical memory subtest of the Rivermead behavioral memory test (42)
	Rey visual design learning test (15 drawings, sum of five trials) (43)
Delayed memory	Delayed recall of the auditory verbal learning test (41)
	Delayed recall of the logical memory (42)
	Delayed recall of the Rey visual design learning test (43)
	Recognition of the Rey visual design learning test (43)
Working memory	Digit span forwards (44)
	Digit span backwards (44)
Executive functioning	Letter cancellation test (time to complete) (41)
	Trail making test (part B subtracted with part A, times to complete) (45)
	Stroop test (word subtest subtracted with color subtest, times to complete) (46)
	Verbal categorical fluency (47)
Processing speed	Trail making test, part A (time to complete) (45)
	Stroop color subtest (time to complete) (46)
Motor dexterity	Finger tapping, right hand (41)
	Finger tapping, left hand (41)

### Risk Factor Assessment

Body mass index and occurrence of high blood pressure, diabetes, and dyslipidemia in the patient groups were recorded. Smoking was assessed in pack-years (smoking years^*^amount of cigarette packs per day). Depressive symptoms were screened at each neuropsychological assessment with the 13-item version of Beck Depression Inventory (48).

### Statistical Analyses

We performed statistical analyses with IBM SPSS 26 (49). *P-*values below 0.05 were considered significant. Neuropsychological test scores were standardized relative to the controls' baseline performance and timed *z-*scores were inverted. Standardized test scores were then averaged within each functional domain. All analyses of cognitive functioning were performed within functional domains.

Differences between symptomatic and asymptomatic CEA patients, between CEA patients operated on the right or left side, and between CEA patients with or without a history of CABG or AMI, were assessed within each functional domain with Mann–Whitney U tests or independent samples *t-*tests for baseline cognitive performance and with χ^2^ tests or Fisher's exact tests for POCD and POCI frequencies. Differences between the control, CEA and CABG groups were assessed with Kruskal–Wallis test for age and χ^2^ tests or Fisher's exact tests for sex, education class and occupation class. Bonferroni corrected *z-*tests for independent proportions were used for *post-hoc* comparison of sex between groups. Differences between the CEA and CABG groups were calculated with Mann–Whitney U tests for smoking and depressive symptoms, independent samples *t-*test for body mass index and χ^2^ tests for diabetes, high blood pressure and dyslipidemia.

We used linear mixed models to assess differences in cognition between groups (CEA, CABG and controls) and interactions in cognition between group and assessment (baseline, acute phase and stable phase). Beforehand, reflection and square root transformation was applied to executive functioning and reflection and logarithmic transformation to processing speed. Age, sex and education class were controlled for in the models. Compound symmetry was chosen for repeated effect covariance structure based on Bayesian information criteria. Significant interactions were assessed *post-hoc* with pairwise comparisons between groups within each measurement. When no significant interactions were found, significant main effects of group were assessed with pairwise comparisons between groups. These *post-hoc* analyses were based on estimated marginal means and Bonferroni corrected.

POCD and POCI were determined based on the reliable change index in order to take into account normal variation and practice effects of the control group (50–52) with a cu*t-*off criterion of ±2. Differences in POCD and POCI frequencies between patient groups were tested with χ^2^ tests or Fisher's exact tests.

We used Bonferroni corrected Friedman tests to assess changes in depressive symptoms between assessments within groups. *Post-hoc* analyses with Wilcoxon signed-rank tests were conducted with a Bonferroni correction applied and r was used as an estimate of effect sizes (53).

## Results

### Baseline Characteristics

Baseline characteristics of the patient and control groups are shown in Table 1. There were no significant differences in age, educational level or occupational level between the control and patient groups (all *p-*values >0.05 in Kruskal–Wallis test and χ^2^ tests), but sex differed significantly between groups (χ^2^(2) = 9.64, *p* = 0.008, χ^2^ test). According to *post-hoc z-*tests, there were no significant differences in sex between controls and either patient group (*p-*values >0.05), but there were significantly more men in the CABG group than in the CEA group (*p* = 0.007).

The CEA group had a longer smoking history than the CABG group (U (142) = 2793.50, *p* = 0.009, Mann–Whitney U test). There were no other differences in cardiovascular risk factors between the patient groups (all *p-*values 0.05 in Mann–Whitney U test, independent samples *t-*test and χ^2^ tests). No patients in the CABG group had a history of earlier CEA. In the CEA group, in total 12 patients had a cardiac history: 9 patients had a previous AMI and 8 patients a previous CABG.

### Differences Within the CEA Group

No clinical strokes were detected in the CEA patients after operation, but two patients showed minor asymptomatic strokes in postoperative MR imaging. Of those patients, one did not show POCD or POCI in postoperative measurements, whereas one exhibited POCD in processing speed at the stable phase.

There were no significant differences between symptomatic and asymptomatic CEA patients in baseline cognitive performance (Mann–Whitney U tests or independent samples *t-*tests) or in POCD or POCI frequencies (χ^2^ tests or Fisher's exact tests) within functional domains (all *p-*values >0.05) in any cognitive domain.

There were no differences between CEA patients operated on the right or left side in baseline cognitive performance in any cognitive domain (*p-*values >0.05 in Mann–Whitney U tests or independent samples *t-*tests). The CEA patients operated on the right side exhibited POCD in motor dexterity significantly more often (25% of patients) than patients operated on the left side (0% of patients) in the stable phase (*p* = 0.018 in Fisher's exact test). Furthermore, the CEA patients operated on the right side showed POCI in executive functioning significantly more often than patients operated on the left side both in the acute phase (right side 30% of patients, left side 4% of patients, *p* = 0.035 in Fisher's exact test) and in the stable phase (right side 50% of patients, left side 13% of patients, *p* = 0.007 in χ^2^ test). There were no other differences in POCD or POCI frequencies between CEA patients operated on the right or left side (*p-*values >0.05 in Fisher's exact tests).

The CEA patients with a history of CABG or AMI performed significantly poorer at baseline in working memory than patients without cardiac history [*t*_(42)_ = 2.40, *p* = 0.02, independent samples *t-*test]. There were no other differences between CEA patients with or without cardiac history in baseline performance (Mann–Whitney U tests or independent samples *t-*tests) or in POCD or POCI frequencies (Fisher's exact tests) in any functional domain (all *p-*values >0.05).

### Differences in Cognitive Performance Between the Control and Patient Groups

**Figure 1:**
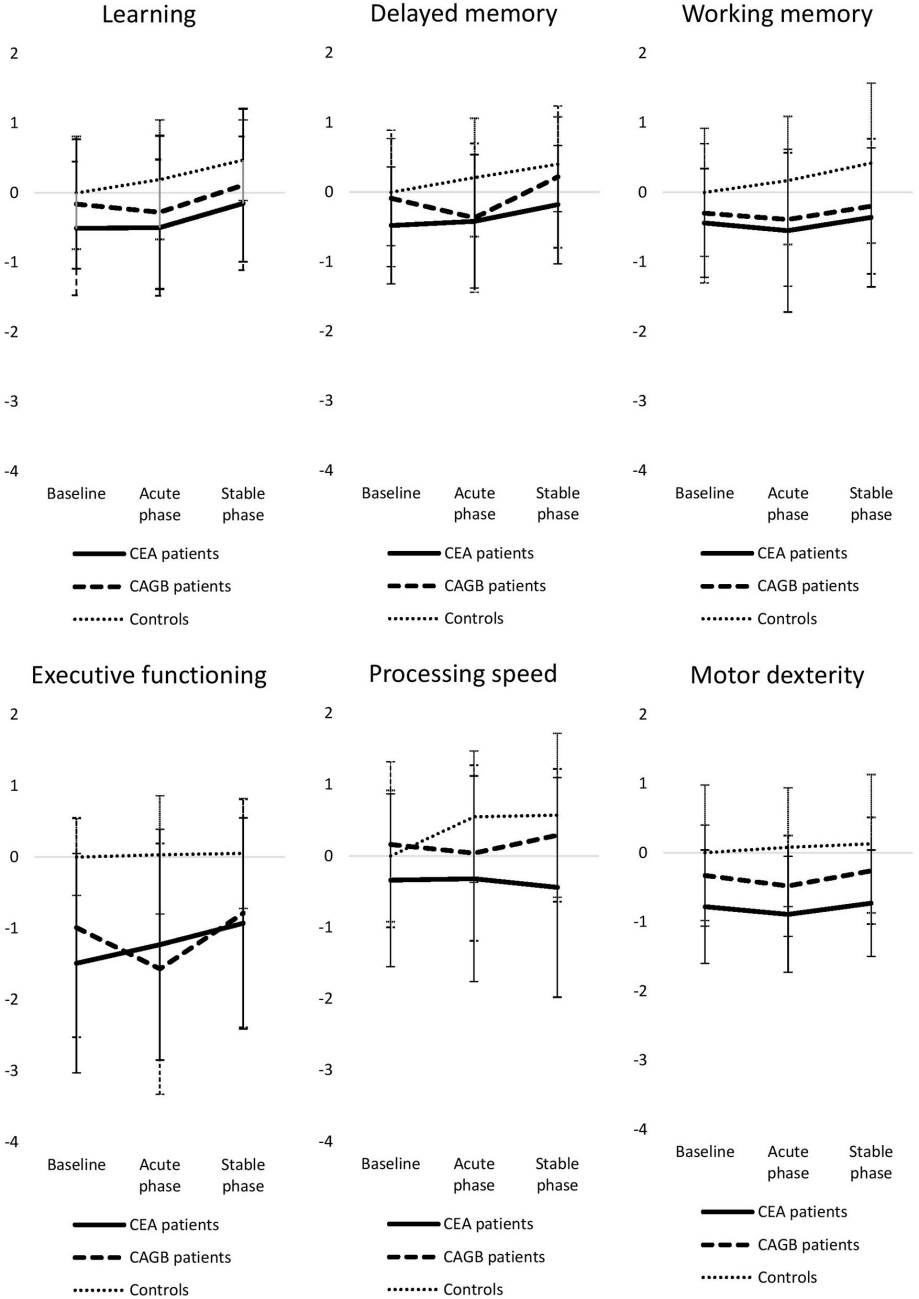
Time-course of groupwise cognitive performance in each functional domain expressed as mean *z-*scores. Error bars reflect standard deviations. CEA, carotid endarterectomy; CABG, coronary artery bypass grafting.

Cognitive performance within groups and cognitive functions is shown in Table 3 and Figure 1. Linear mixed models showed an interaction between group (CABG, CEA and control) and measurement (baseline, acute phase and stable phase) in delayed memory [*F*_(4,314.13)_ = 5.17, *p* < 0.001], executive functioning [*F*_(4,315.17)_ = 4.83, *p* < 0.001] and processing speed [*F*_(4,314.85)_ = 5.02, *p* < 0.001].

**Table 3:** Cognitive performance of patients and controls in each measurement.

**Functional domain**	**CEA patients**			**CABG patients**			**Controls**		
	**Baseline**	**Acute phase**	**Stable phase**	**Baseline**	**Acute phase**	**Stable phase**	**Baseline**	**Acute phase**	**Stable phase**
Learning	−0.51 ± 0.96	−0.50 ± 0.98	−0.15 ± 0.96	−0.16 ± 0.93	−0.28 ± 1.10	0.11 ± 1.10	0 ± 0.81	0.19 ± 0.86	0.47 ± 0.58
Delayed memory	−0.48 ± 0.84	−0.42 ± 0.96	−0.18 ± 0.85	−0.09 ± 0.98	−0.37 ± 1.07	0.22 ± 1.02	0 ± 0.77	0.21 ± 0.85	0.40 ± 0.68
Working memory	−0.44 ± 0.78	−0.55 ± 1.17	−0.36 ± 1.00	−0.30 ± 1.00	−0.39 ± 0.96	−0.20 ± 0.97	0 ± 0.92	0.17 ± 0.92	0.42 ± 1.15
Executive functioning	−1.49 ± 1.54	−1.23 ± 1.62	−0.93 ± 1.48	−0.99 ± 1.54	−1.57 ± 1.76	−0.79 ± 1.60	0 ± 0.54	0.03 ± 0.83	0.05 ± 0.77
Processing speed	−0.34 ± 1.21	−0.32 ± 1.44	−0.44 ± 1.51	0.16 ± 1.16	0.04 ± 1.23	0.29 ± 0.93	0 ± 0.92	0.55 ± 0.60	0.57 ± 0.57
Motor dexterity	−0.78 ± 0.82	−0.89 ± 0.84	−0.73 ± 0.77	−0.33 ± 0.73	−0.48 ± 0.73	−0.26 ± 0.77	0 ± 0.98	0.08 ± 0.86	0.13 ± 1.00

*Unadjusted, untransformed mean z-scores ± SD standardized relative to the baseline of controls. CEA, carotid endarterectomy; CABG, coronary artery bypass grafting*.

*Post-hoc* analyses of significant interactions are reported as follows. *At baseline*, the CEA group performed significantly worse than controls in executive functioning [*t*_(256.01)_ = 0.38, *p* = 0.003] and significantly worse than the CABG group in processing speed [*t*_(232.28)_ = 0.23, *p* = 0.02]. *In the acute phase*, the CABG group performed worse than controls in delayed memory [*t*_(213.67)_ = 0.62, *p* = 0.02] and in executive functioning [*t*_(253.38)_ = 0.41, *p* < 0.001]. The CEA group performed worse than controls in executive functioning [*t*_(256.01)_ = 0.33, *p* = 0.01] and in processing speed [*t*_(238.55)_ = 0.37, *p* = 0.01]. *In the stable phase*, the CEA group performed worse than both the CABG group [*t*_(232.77)_ = 0.28, *p* = 0.003] and controls [*t*_(238.55)_ = 0.42, *p* = 0.004] in processing speed. Please note that changes in *post-hoc* tests of transformed variables, executive functioning and processing speed, are not absolute but proportional.

There were no interactions between group and measurement in the other functional domains, but there was a significant main effect of group in motor dexterity (F(2, 153.58) = 10.79, *p* < 0.001). *Post-hoc* analyses showed that both the CEA group [*t*_(153.60)_ = 0.82, *p* < 0.001] and the CABG group [*t*_(153.02)_ = 0.59, *p* = 0.001] differed significantly from controls in motor dexterity. There were no significant differences between groups in learning and working memory.

### POCD and POCI Within the Patient Groups

Frequencies of POCD and POCI are shown in Table 4. According to χ^2^ and Fisher's exact tests, the CABG patients exhibited POCD in the acute phase significantly more frequently than the CEA patients in most functional domains. In the stable phase there were no significant group differences in POCD frequencies. In contrast, POCI in executive functioning was more frequent in the CEA group than the CABG group in the acute phase, but the difference between groups did not reach significance in the stable phase. There were no differences in POCI frequency in other functional domains.

**Table 4:** Cognitive changes at the acute and stable phase.

	**Acute phase**			**Stable phase**		
	**CEA patients**	**CABG patients**	***p-*value**	**CEA patients**	**CABG patients**	***p-*value**
POCD in any domain	10 (23%)	65 (65%)	0.000^***^	15 (34%)	46 (46%)	0.183
Learning	3 (7%)	20 (20%)	0.047^*^	5 (11%)	20 (20%)	0.199
Delayed memory	1 (2%)	21 (21%)	0.004^**^	3 (7%)	6 (6%)	1.000
Working memory	2 (5%)	8 (8%)	0.724	2 (5%)	13 (13%)	0.149
Executive functioning	5 (11%)	37 (37%)	0.002^**^	3 (7%)	14 (14%)	0.212
Processing speed	2 (5%)	19 (19%)	0.024^*^	6 (14%)	6 (6%)	0.189
Motor dexterity	1 (3%)	12 (12%)	0.111	5 (12%)	11 (11%)	1.000
POCI in any domain	9 (21%)	9 (9%)	0.056	17 (39%)	30 (30%)	0.309
Learning	1 (2%)	1 (1%)	0.519	1 (2%)	2 (2%)	1.000
Delayed memory	2 (5%)	0 (0%)	0.093	0 (0%)	4 (4%)	0.312
Working memory	0 (0%)	2 (2%)	1.000	1 (2%)	2 (2%)	1.000
Executive functioning	7 (16%)	4 (4%)	0.035^*^	13 (30%)	18 (18%)	0.128
Processing speed	1 (2%)	2 (2%)	1.000	1 (2%)	2 (2%)	1.000
Motor dexterity	0 (0%)	1 (1%)	1.000	2 (5%)	6 (6%)	1.000

*Data are presented as N (%). p-values are from χ^2^ tests and Fisher's exact tests*.

* *p < 0.05*,

** *p < 0.01*,

*** *p < 0.001*.

*POCD, postoperative cognitive dysfunction; POCI, postoperative cognitive improvement, both determined with the reliable change index. CEA, carotid endarterectomy; CABG, coronary artery bypass grafting*.

### Mood

Depressive symptoms in each group and assessment are shown in Table 1. There were no significant differences in depressive symptoms between the patient groups in any assessments (all *p-*values >0.05 in Mann–Whitney U tests). There was a significant change in mood between assessments in the CABG group (χ^2^ (2) = 32.03, *p* < 0.001) but not in the control or CEA groups (*p-*values >0.05, Friedman tests). According to *post-hoc* analyses, depressive symptoms in the CABG group were significantly more pronounced in the acute phase than at baseline (*Z* = –3.27, *p* = 0.010, *r* = –0.23), whereas depressive symptoms were lower in the stable phase compared with baseline (*Z* = –3.43, *p* = 0.006, *r* = –0.24) and with the acute phase (*Z* = –4.53, *p* < 0.001, *r* = –0.33 in Wilcoxon signed-rank tests).

## Discussion

The present study was set to explore cognitive differences between patients receiving surgical treatment for stenosing atherosclerotic changes in either carotid or coronary arteries. The results show that carotid patients exhibited more pronounced cognitive deficits than healthy controls or coronary artery patients before treatment in the domains of executive functioning and processing speed. Following surgery, CABG patients showed more frequent short-term postoperative decline than CEA patients, indicating that POCD is a heterogeneous condition. Furthermore, postoperative cognitive improvement in executive functioning was more pronounced in CEA patients than CABG patients. Thus, despite worse presurgical cognitive level, CEA patients seemed to gain more cognitive benefit from surgical treatment than CABG patients.

Before surgical treatment, the CEA patients in our study performed worse than controls in executive functioning and worse than CABG patients in processing speed, whereas no differences were detected in other functional domains. The difference between CABG patients and controls was not significant. However, the lack of significance was probably due to a low-power setting as we have previously shown with the same dataset that also CABG patients perform worse than controls at baseline (10). Nevertheless, the prominent finding is the more pronounced presurgical cognitive deficit in several domains in CEA patients compared with CABG patients, despite the overall relative similarity of the patient groups and their risk profiles. Considering the fact that all CEA patients had a high-grade stenosis, the finding lends support to the hypothesis of hypoperfusion of the anterior cerebrum playing a notable role in the cognitive impairment, along with risk factors and the general vascular disease. The hypothesis is further supported by the change in executive functions, whose network is to a great extent subserved by frontal lobes (54), supplied by the carotid arteries. In contrast, patients suffering from coronary artery disease exhibit more general deficit with low-output circulation and a more global cerebral hypoperfusion (2). Our results suggest that these factors cause generally less cognitive deterioration whereas carotid disease *per se* has a greater propensity to affect cognition, especially executive functioning and processing speed, in comparison with coronary disease.

The postoperative cognitive paths of CABG and CEA patients were clearly divergent. On group level, the CABG patients exhibited cognitive decline of delayed memory and executive functioning in the acute phase after surgery, but the decline was no longer detected in a more stable phase of recovery, 3 months after surgery. Similar results were found on an individual level: the CABG patients exhibited POCD significantly more often than the CEA patients in most functional domains (learning, delayed memory, executive functioning and processing speed) in the acute phase but not in the stable phase. In contrast, the CEA patients did not show group*-*level decline in the acute phase. In the stable phase, they remained inferior to the CABG patients in processing speed and also performed worse than healthy controls, indicating a slight deterioration or lower learning ability. However, on an individual level, the CEA patients did not show more marked POCD than the CABG patients in processing speed or in any other functional domain in either postoperative phase. Thus, the findings support the greater risk of cognitive decline in the context of CABG than CEA. The more general character of the decline following CABG tallies with the presence of systemic factors such as cerebral perfusion changes, microembolization or inflammatory reactions (23–25), which all may contribute to a more serious stress to the brain. In addition, postoperative pain and sleep disturbances related to major cardiac surgery could have an effect on cognitive performance shortly after operation (23, 25) and thus explain some of the observed differences in cognition between the CABG and CEA group at the acute phase. The cognitive decline in CABG patients, however, tends to be reversible, as reported also previously with the very same dataset (10). Ultimately, the results underline the fact that POCD is a heterogeneous condition.

Postoperative cognitive improvement was detected in CEA patients in executive functioning. On group level, some improvement was observable already shortly after surgery, and it was more marked in the stable phase, at which time the CEA patients no longer differed significantly from healthy controls. Also on an individual level, POCI of executive functioning in the CEA group was more frequent in the stable phase than in the acute phase.

Generally, earlier studies have not found differences related to side of CEA in postoperative cognitive functioning (16, 17, 55, 56). However, in a more detailed domain-specific analysis we were able to show that POCI of executive functioning was significantly more frequent in the CEA patients operated on the right than on the left side in both postoperative assessments. This finding however likely attributes to the chosen test methods that tap various aspects of executive functioning, which have been shown to recruit differential prefrontal regions both on the left and right side of the brain (57–59).

Comparing between the patient groups, POCI in executive functioning was significantly more frequent within the CEA than the CABG group in the acute phase (16% in CEA patients, 4% in CABG patients), but the difference in frequency in the stable phase (30% in CEA patients, 18% in CABG patients) was no longer significant. These findings suggest that while carotid patients show worse preoperative performance than coronary patients in executive functions, probably due to the hypoperfusion in the anterior circulation, they also benefit more from surgical treatment. Indeed, earlier studies have shown that recovery of cerebral circulation after carotid revascularization is related to improved cognition (31). However, although the CEA patients in our study improved gradually in time, resulting in POCI in as many as roughly a third of patients in the stable phase after surgery, the difference in frequency of POCI between CEA and CABG patients decreased in time. This finding can be explained by recovery from short-term postoperative deterioration in the CABG group.

There were also differences in postoperative changes in mood between the patient groups. In accordance with previous findings (60) we found no changes in depressive symptoms after CEA. In contrast, there were changes in mood after CABG in the present study: depressive symptoms were significantly higher in the acute phase and reduced in the stable phase compared with baseline. These results are consistent with earlier findings (61) and may be explained by a drop in general health shortly after major operation following with positive long-term effects of CABG on general health. However, improved mood in the stable phase after CABG was not accompanied with better cognitive functioning in our study, whereas CEA was associated with improved cognition despite no significant changes in mood. Hence, the observed differential cognitive trajectories after CEA and CABG do not seem to attribute to changes in mood.

This is the first study to compare cognitive functioning between atherosclerotic patients undergoing CEA or CABG, to the best of our knowledge. The study has several strengths, such as an extensive neuropsychological test battery organized in several functional domains and estimation of POCD and POCI in relation to learning effects and normal variation of a control group. There are, however, some limitations, most importantly the restricted sample size resulting in reduced statistical power. Indeed, some previously reported differences between the CABG group and controls with the same data (10) did not reach significance in this study. Considering the restrictions in sample size, the clear differences between the patient groups in the present study indicate a rather robust finding.

One limitation is that no direct measure for the cerebral hypoperfusion was obtained for this study. Still, only high-grade carotid stenoses were recruited in the CEA group, which makes the existence of hypoperfusion probable in this group. Also, the presence of delirium was not assessed in either patient group and its potential influence on cognitive changes in the stable phase could not be assessed. Another potential limitation is that there was no similar assessment of carotid arteries in the CABG patients or coronary arteries in the CEA patients. The CABG patients did undergo a screening carotid ultrasound to preclude a hemodynamically significant stenosis, which makes the co-existence of severe carotid stenosis highly improbable in the CABG group. While we cannot exclude the possibility that some CABG patients had a clinically insignificant carotid stenosis that could still have some effect on their cognitive performance (62), our findings suggest that the cognitive trajectories of atherosclerotic patients are different in patients with a primary symptom in carotid or coronary artery. Similarly, we cannot exclude that some CEA patients also had stenoses in coronary arteries; atherosclerotic disease is a systemic disease with an effect on the various vascular beds of the body. The minority of the CEA group that indeed had a history of earlier cardiac disease performed worse than the other CEA patients at baseline in working memory, but there were no significant differences in other functional domains. Thus, a more severe atherosclerotic disease does not seem to explain preoperative differences between groups in executive functioning and processing speed. Furthermore, there were no differences between CEA patients with or without cardiac history in POCD or POCI frequencies in any functional domain, suggesting that the severity of atherosclerotic disease did not account for the detected postoperative differences in cognition between the patient groups. Neither were there any differences in cardiovascular risk factors between the CEA and CABG groups, apart from longer smoking history in the CEA than the CABG group. Despite the more frequent smoking and the potential of a more severe atherosclerotic disease, the CEA group showed less postoperative cognitive deterioration and greater improvement than the CABG patients. Hence, the different cognitive trajectories of the CEA and CABG groups do not seem attributable to the severity of atherosclerotic disease.

A further limitation in this study is that the CEA group included both symptomatic (TIA or minor stroke) and asymptomatic patients. It could be argued that inferior preoperative cognitive performance or more marked postoperative improvement in the CEA group compared with the CABG group would be related to cognitive symptoms of recent stroke and subsequent spontaneous recovery of stroke symptoms. The detected strokes in the symptomatic group were, however, minor with no need for inpatient rehabilitation, as defined by inclusion criteria, and there were no significant differences between symptomatic and asymptomatic CEA patients in either baseline cognitive performance or frequencies of POCD or POCI in any functional domain. Thus, our results suggest that the differences between CEA and CABG group are not related to strokes in the CEA group but are, indeed, markers for differential cognitive trajectories between carotid and coronary stenosis patients.

In conclusion, the present study suggests that the cognitive effects of atherosclerosis are different in CEA and CABG patients, with a greater and more focal effect in CEA patients. Furthermore, surgical treatment of carotid and coronary artery disease leads to differential cognitive paths. CEA patients exhibit worse presurgical cognitive status but seem to gain more from surgical treatment than CABG patients. CABG patients, in contrast, show postoperative cognitive decline more frequently than CEA patients, but it is mostly transient. Our results indicate that POCD should not be considered as a unified phenomenon: instead, it is heterogeneous and dependent on type of revascularization surgery. In addition, our results suggest that carotid artery patients are at a greater risk for cognitive decline than coronary patients preoperatively but may gain more cognitive benefit from surgery.

## Data Availability Statement

The datasets presented in this article are not readily available due to ethical restrictions. Requests to access the datasets should be directed to kristiina.relander@helsinki.fi.

## Ethics Statement

The studies involving human participants were reviewed and approved by the Ethical Committee of the Helsinki University Hospital, Finland. The patients/participants provided their written informed consent to participate in this study. Requests for sharing of data will be given individual consideration after additional approval for sharing by the local Ethics Committee.

## Author Contributions

RR, PL, KR, LS, and MH contributed to the conception and design of the study. The data acquisition was designed and put into practice by RR, IT, AV, JR, LS, and MH. The data analysis was performed by KR who also drafted the article manuscript, which was critically revised by RR, PL, MH, and LS. All authors discussed the analyses, commented on the manuscript, and approved the final submitted version of the manuscript.

## Funding

The study was supported by the Helsinki University Hospital governmental subsidy funding for clinical research. KR was funded by the Alfred Kordelin foundation and Helsinki University Hospital governmental subsidy funding for clinical research.

## Conflict of Interest

The authors declare that the research was conducted in the absence of any commercial or financial relationships that could be construed as a potential conflict of interest.

## Publisher's Note

All claims expressed in this article are solely those of the authors and do not necessarily represent those of their affiliated organizations, or those of the publisher, the editors and the reviewers. Any product that may be evaluated in this article, or claim that may be made by its manufacturer, is not guaranteed or endorsed by the publisher.
